# The importance of prebiotics in managing colic in horses: focus on *Akkermansia muciniphila* and its anti-inflammatory potential

**DOI:** 10.3389/fvets.2026.1759381

**Published:** 2026-02-24

**Authors:** Ashley Cottone, Keely Seiter, Brinley Thomas, Nathan Schank, Michelle Wulf, Lynda Miller, Stacy Anderson, Undral Munkhsaikhan, Ashutosh Verma, Ammaar H. Abidi, Modar Kassan

**Affiliations:** 1 College of Dental Medicine, Lincoln Memorial University, LMU Tower, Knoxville, TN, United States; 2 Richard A. Gillespie College of Veterinary Medicine, Lincoln Memorial University, Harrogate, TN, United States

**Keywords:** *Akkermansia muciniphila*, equine colic, inflammation, microbiome, prebiotics

## Abstract

Colic remains one of the most frequent and costly causes of equine morbidity and mortality, with significant welfare and economic implications. Disturbances in the gut microbiome are increasingly recognized as an important contributing factor. In recent years, prebiotics, non-digestible substrates that promote beneficial microbes, have emerged as promising microbiome-targeted strategies. *Akkermansia muciniphila* (*A. muciniphila*) has gained attention for its unique ability to degrade mucin, maintain epithelial integrity, and exert potent anti-inflammatory effects. Although its benefits are well established in humans and rodent models, little is known about its abundance, function, and therapeutic potential in horses. This review evaluates current evidence on prebiotics and *A. muciniphila* in equine gut health and outlines their translational potential by examining biological mechanisms, feasibility of therapeutic application, and implications for equine colic prevention. Importantly, this review is intended as a hypothesis-generating synthesis rather than evidence of causality. Proposed mechanisms and therapeutic implications are based primarily on extrapolation from non-equine models and limited equine observational data, highlighting critical knowledge gaps and the need for controlled, hypothesis-driven studies in horses.

## 1 Introduction

Equine colic is a clinical syndrome defined by abdominal pain that can stem from a broad spectrum of gastrointestinal disturbances, ranging from transient gas accumulation to severe and life-threatening intestinal strangulation (1). Colic is consistently recognized as the most common emergency in equine practice and remains a leading cause of mortality worldwide (2). Recent epidemiological reports estimate approximately 3–10 colic episodes per 100 horses annually, with case-fatality rates ranging from 6 to 15% in severe forms (3–5). These clinical and economic implications underscore the need for improved understanding of risk factors and pathophysiology.

The gastrointestinal microbiota plays a central role in both the pathogenesis and prevention of colic (6). The gastrointestinal microbiota plays an important role in the pathophysiology of certain forms of equine colic, particularly those classified as medical colic. Medical colic encompasses functional and inflammatory gastrointestinal disorders, including dysmotility, hindgut acidosis, gas accumulation, and immune-mediated intestinal dysfunction. In these contexts, disruptions of microbial balance (dysbiosis), impaired fermentation, and compromised mucosal barrier function are increasingly recognized as contributing factors. In contrast, surgical colic arises from mechanically driven events such as strangulation, volvulus, entrapment, or obstruction and is not initiated by microbiome alterations, although secondary dysbiosis and barrier disruption may exacerbate systemic inflammation following ischemic injury.

Horses, as hindgut fermenters, depend extensively on their intestinal microbial community for fiber fermentation, short-chain fatty acid (SCFAs) production, and sustaining the majority of their metabolic energy supply (7). This community is a highly diverse ecosystem composed of bacteria, archaea, fungi, protozoa, and viruses, often referred to as a “forgotten organ” because of its deep integration with host metabolism, mucosal immunity, and barrier function (8). In humans and other animals, disruptions of microbial balance, termed dysbiosis, have been implicated in a wide range of diseases, including asthma, inflammatory bowel disease, obesity, and colorectal cancer (9). In horses, dysbiosis is increasingly recognized as a key contributor to colic risk, although equine-specific mechanistic studies remain limited (10).

Therapeutic strategies aimed at preserving or restoring microbial balance are therefore gaining attention in equine medicine (10). Prebiotics, defined as selectively fermentable substrates that promote the growth of beneficial microbes, are of particular interest (11). Prebiotics may improve gut resilience by enhancing SCFAs production, supporting epithelial integrity, and modulating immune responses (12). Recent studies have highlighted the potential of novel prebiotics, including complex polysaccharides like Levan, to modulate gut ecology in ways that could improve gastrointestinal resilience in horses (10, 13).

Among emerging microbial targets*, Akkermansia muciniphila* (*A. muciniphila*) has attracted considerable attention. This mucin-degrading bacterium, abundant in the gut lining, is increasingly recognized as a keystone species due to its role in maintaining mucosal barrier integrity, regulating host metabolism, and producing metabolites that influence immune homeostasis. In human studies, reduced abundance of *A. muciniphila* has been linked to obesity, diabetes, and inflammatory disorders (14). While equine-specific data are still sparse, preliminary findings suggest that *A. muciniphila* may also contribute to gut stability in horses and may potentially serve as a biomarker or therapeutic target for colic prevention (15, 16). It is important to note that the limited equine studies identifying reduced *A. muciniphila* in horses with colic report this change as part of a broader dysbiotic profile involving multiple bacterial families, rather than as an isolated finding. These data suggest that alterations in *A. muciniphila* abundance may reflect overall microbial instability rather than a single causal mechanism in colic pathophysiology.

**Figure 1:**
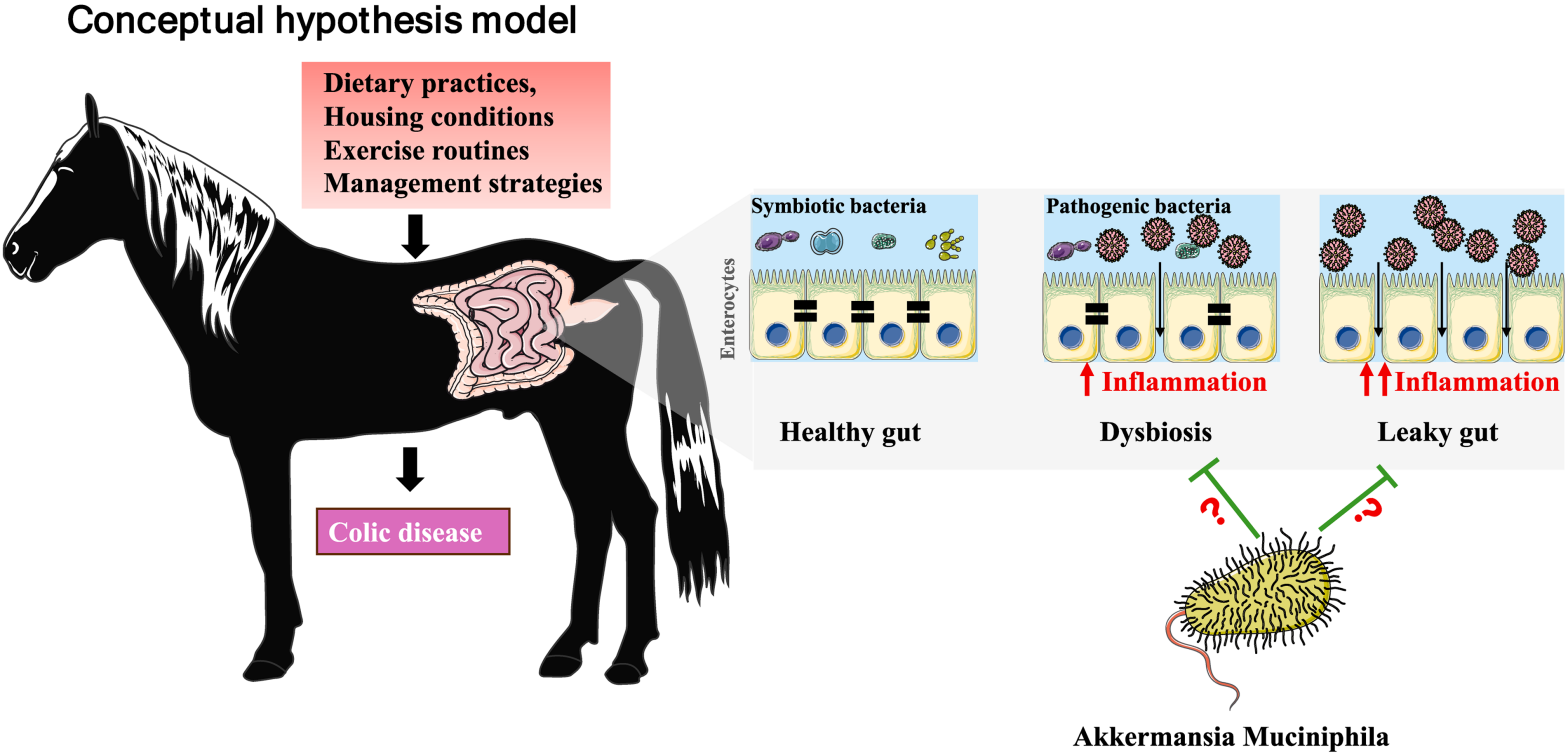
The impact of management factors on equine gut health and colic risk. This figure presents a conceptual model outlining the pathway through which common equine management factors influence gut health and colic risk. The model proposes that key inputs, including dietary practices, housing conditions, exercise routines, and management strategies, can disrupt gut homeostasis by triggering two interconnected pathological processes: first, by inducing dysbiosis, an imbalance between beneficial (symbiotic bacteria) and harmful (pathogenic bacteria) microbial populations, and second, by promoting inflammation that compromises the intestinal barrier, resulting in a leaky gut. The convergence of these two states, dysbiosis and a leaky gut, creates a pathological environment that disrupts the healthy gut and significantly elevates the risk for the development of colic disease. As a mucin-degrading, barrier-supporting bacterium, *A. muciniphila* helps maintain epithelial integrity, reduces inflammation, and stabilizes microbial composition. Its presence in the model indicates that loss or reduced abundance of *A. muciniphila* may exacerbate dysbiosis and barrier dysfunction, while its restoration through targeted prebiotic strategies could counteract these mechanisms and lower colic risk. Question marks indicate areas where causality has not been established. Dysbiosis and intestinal barrier dysfunction may precede colic, result from colic, or coexist as part of a bidirectional process. *A. muciniphila* is shown as a putative modulatory component of mucosal homeostasis based on mechanistic evidence from non-equine models; however, its protective role in equine colic has not been demonstrated. This figure is intended to highlight biological plausibility and knowledge gaps rather than depict a linear or causal pathway. This conceptual model is most applicable to medical colic associated with dysbiosis, inflammation, and barrier dysfunction and does not represent the initiating mechanisms of surgical colic.

This review synthesizes current evidence on the equine gut microbiota, focusing on its role in colic pathophysiology and potential avenues for modulating microbiota. We first outline the normal composition and function of the equine intestinal microbiome, then examine factors that disrupt microbial homeostasis and contribute to dysbiosis. Finally, we explore therapeutic approaches, particularly prebiotic interventions and their potential to enhance *A. muciniphila* abundance, as promising strategies for improving equine gut health and reducing the burden of colic across diverse equestrian populations. Figure 1 illustrates the conceptual relationship between dysbiosis, mucosal barrier disruption, and the potential stabilizing role of *A. muciniphila* in mitigating colic-related pathophysiology (Figure 1). The purpose of this review is not to establish a causal role for prebiotics or *A. muciniphila* in equine colic, but rather to integrate existing mechanistic, translational, and limited equine data into a coherent biological framework. The hypotheses presented are intended to identify plausible pathways, inform study design, and highlight areas where direct equine research is urgently needed.

Accordingly, microbiome-targeted strategies discussed in this review are intended to address medical forms of colic rather than mechanically driven surgical colic.

## 2 Prebiotics and equine gut health

Throughout this section, mechanistic insights from human and rodent studies are discussed to establish biological plausibility; however, these findings should not be interpreted as evidence of efficacy in adult horses unless explicitly supported by equine data.

Prebiotics, which selectively stimulate beneficial gut microbes, have been particularly explored in horses for their ability to stabilize hindgut fermentation and microbial balance (11, 17, 18). In healthy horses, the hindgut microbiome is dominated by Firmicutes and Bacteroidetes, with contributions from Verrucomicrobia, Proteobacteria, and Fibrobacteres (6, 19). These microbial communities support extensive fiber fermentation, maintain epithelial integrity, produce essential metabolites such as SCFAs, and regulate immune–mucosal homeostasis (10, 20). Understanding this normal baseline ecology is essential for interpreting how prebiotics modulate equine gastrointestinal function. In horses, the most extensively studied prebiotics include fructooligosaccharides (FOS), mannan-oligosaccharides (MOS), and inulin (21). These carbohydrates are resistant to enzymatic digestion in the foregut, allowing them to reach the cecum and colon intact, where they become fermentable substrates for the hind gut microbiota (22). Importantly, equine-specific data demonstrating a direct increase in *A. muciniphila* following prebiotic supplementation are currently limited. The only study reporting increased *A. muciniphila* abundance following oligosaccharide supplementation was conducted in foals at day 49 of life, whereas no such increase was observed in adult mares receiving fructooligosaccharides (FOS) or mannan-oligosaccharides (MOS). These findings reflect responses observed in a younger animal. Because data from mature mares are not available, it remains unclear whether similar responses occur in adult horses.

The fermentation process generates SCFAs, notably acetate, propionate, and butyrate, which serve multiple functions (23). Acetate is a major energy source for peripheral tissues, propionate supports gluconeogenesis, and butyrate fuels colonocytes while also exerting anti-inflammatory effects (24). The production of SCFAs also lowers luminal pH, creating an environment less favorable to pathogenic bacteria such as *Clostridium difficile* and *Salmonella* (11).

Prebiotics exert their benefits not only by promoting the growth of well-known beneficial genera, such as *Lactobacillus* and *Bifidobacterium*, but also potentially by fostering the growth of less abundant yet highly influential species, including *A. muciniphila*. These taxa exert outsized influence through their roles in mucin turnover, barrier maintenance, and host immune regulation, making them key contributors to gut homeostasis (22, 25). Through cross-feeding interactions, certain prebiotics can indirectly enhance the ecological niche for these microbes, supporting mucin degradation and mucus layer renewal (25). However, extrapolating findings from monogastric species to hindgut fermenters such as horses should be approached with caution, as horses exhibit distinct mucin composition, unique fermentation dynamics, and a different microbial niche structure within the large intestine.

Clinical observations in equine populations have shown that prebiotic supplementation can stabilize hindgut microbial composition during abrupt feed changes, improve fecal consistency, and reduce signs of digestive upset (26). Horses receiving MOS or FOS have demonstrated higher total SCFAs concentrations in fecal samples and a reduced presence of pathogenic bacteria (10). These benefits are particularly relevant during high-risk periods such as post-antibiotic recovery, transport, or changes from pasture to stall confinement (27). However, responses can be highly individual, influenced by baseline microbiota composition, diet, and host factors, highlighting the need for precision approaches rather than one-size-fits-all supplementation. Importantly, equine-specific dose–response studies defining optimal or adverse thresholds for prebiotic supplementation—particularly with respect to targeting *A. muciniphila*, are currently lacking. Available equine studies using fermentable fibers and prebiotic compounds typically employ doses in the range of approximately 0.1–0.5 g/kg body weight per day, though these studies were not designed to establish safety margins or upper limits and should therefore be interpreted cautiously (28165863).

In horses with colic, significant alterations in the gut microbiota are observed, including a reduction in *A. muciniphila*, a genus within the *Verrucomicrobia phylum* that is generally considered beneficial for maintaining intestinal barrier integrity and metabolic balance. The study by Park et al. (15) showed that horses with small intestinal colic had a notably lower abundance of *A. muciniphila* compared to healthy controls, suggesting that loss of this bacterium may contribute to microbial dysbiosis and impaired gut health. Since *A. muciniphila* is associated with mucin degradation, regulation of host immune responses, and protection against pathogen overgrowth in other species, its depletion in colic horses may exacerbate intestinal inflammation and compromise epithelial barrier function. These findings highlight *A. muciniphila* as a potentially important biomarker of equine gut health and a possible therapeutic target for preventing or mitigating colic through microbiota-directed interventions such as prebiotics or dietary modulation. Prebiotic Types Relevant to Equine Gut Health are summarized in Table 1. In horses with colic, significant alterations in the gut microbiota have been reported, including reduced relative abundance of *A. muciniphila* alongside increases in lactic-acid–producing bacteria and streptococci, reflecting a broader dysbiotic state rather than a single taxon-specific change.

**Table 1:** Prebiotic types relevant to equine gut health.

Prebiotic type	Species studied	Main outcomes	Evidence level	Key supporting references
Fructooligosaccharides (FOS)	Horses, humans, other monogastrics	Increases SCFAs production; improves hindgut fermentation; stabilizes microbial composition during dietary changes.	Moderate	Heaton et al. (18), MacNicol et al. (26), Davani-Davari et al. (11), and Yoo et al. (13)
Mannan-oligosaccharides (MOS)	Horses, poultry, pigs, livestock	Reduces pathogen adhesion; modulates immunity; supports hindgut stability during stress.	Strong	Heaton et al. (18), MacNicol et al. (26), Ravanal et al. (21), and Boucher et al. (10)
Inulin/Inulin-type fructans	Horses, humans, rodents	Increases butyrate and SCFAs; supports barrier function; may enhance *Akkermansia* abundance.	Moderate	MacNicol et al. (26), Pérez-Monter et al. (66), Davani-Davari et al. (11), and Yoo et al. (13)

Summary of major prebiotic types relevant to equine gut health, including the primary species in which each has been studied, key microbiological outcomes, and representative supporting references. Evidence levels reflect the strength and specificity of available data, with MOS and FOS supported by both equine and livestock studies, and inulin supported by limited equine data but strong mechanistic evidence from human and rodent research.

Additionally, the study by Park et al. (15) employed a cross-sectional analysis of fecal microbiota collected at the time of hospital admission, with no longitudinal or pre-disease sampling, limiting the ability to infer whether reductions in *A. muciniphila* preceded intestinal disease or occurred secondary to colic-associated dysbiosis. Notably, decreased *A. muciniphila* abundance was observed specifically in horses with small intestinal colic (*n* = 8), a relatively small and clinically heterogeneous subgroup, and occurred alongside broad alterations in microbial diversity, lactic acid–producing taxa, and methanogenic populations. In addition, fecal samples were used as a proxy for hindgut microbial activity, and dietary history prior to admission was not standardized across study groups. Consequently, the observed reduction in *A. muciniphila* should be interpreted as an associative finding within acute intestinal disease rather than evidence of a causal role in colic pathogenesis.

## 3 *Akkermansia muciniphila*: anti-inflammatory and barrier-protective roles

*A. muciniphila* occupies a unique ecological niche within the gut microbiota (28). Rather than competing for dietary fiber like most gut symbionts, it specializes in degrading host-derived mucin glycoproteins in the mucus layer lining the intestinal epithelium (29). At first glance, this might seem counterintuitive; breaking down the mucus barrier would seem to compromise gut integrity (30). However, under physiological conditions *A. muciniphila*–mediated mucin degradation is tightly regulated and stimulates compensatory mucin synthesis; yet, in states of severe dysbiosis or nutrient imbalance, excessive mucin turnover could theoretically thin the mucus layer and increase epithelial vulnerability. In healthy settings, this activity stimulates the host to produce new mucins, which maintain a thick, well-structured, and functionally protective mucus layer over time. In parallel, the breakdown of mucin glycans by *A. muciniphila* yields SCFAs, including acetate, propionate, and succinate, which reinforce epithelial tight junctions, reduce inflammation, and serve as energy sources for colonocytes (23, 24). Additionally, the release of glycan fragments supports cross-feeding of other commensal microbes, enhancing microbial diversity and ecosystem stability. Overall, these activities position *A. muciniphila* as a key regulator of mucosal homeostasis and microbial ecosystem structure (28).

In both human and rodent studies, higher levels of *A. muciniphila* have been inversely linked with obesity, type 2 diabetes, metabolic syndrome, and inflammatory bowel disorders (22). Mechanistically, it appears to exert its influence on the immune system through enhancing the release of anti-inflammatory cytokines such as IL-10 and simultaneously suppressing pro-inflammatory mediators like TNF-*α* and IL-6 (31). However, while these mechanistic insights support biological plausibility, there is currently no evidence that prebiotic supplementation reliably enhances *Akkermansia muciniphila* abundance in adult horses.

The paper by Ludwig et al. identifies Interleukin-6 (IL-6) as a primary cytokine stimulator of the acute phase response and a promising diagnostic and prognostic biomarker for ischemic or inflammatory causes of equine colic (32). Several studies cited in the review demonstrate that IL-6 levels are significantly higher in the blood and peritoneal fluid of horses with strangulating intestinal lesions compared to those without strangulating lesions (33–35). Furthermore, IL-6 concentrations were found to be most elevated in horses affected by ischemic and inflammatory intestinal diseases and were predictive of poor outcomes and correlated with mortality (33–35). The paper concludes that measuring both serum and peritoneal fluid IL-6 levels shows considerable promise as a valuable tool for diagnosing and prognosing severe, inflammatory colic cases in horses. Mechanistically, IL-6 is rapidly released from macrophages, endothelial cells, and damaged enterocytes in response to ischemia and barrier disruption. It stimulates hepatic acute-phase protein synthesis, recruits neutrophils, and amplifies systemic inflammation. In equine colic, particularly with strangulating lesions, this surge likely reflects both local necrosis and endotoxin leakage, making IL-6 a sensitive marker of disease severity and a potential driver of poor outcomes.

Evidence establishes a direct link between TNFα and the systemic inflammatory response in horses with colic (36). It reports that marked increases in circulating TNFα have been specifically linked to a higher rate of mortality in cases of equine colic attributable to gastrointestinal tract disease (37, 38). This finding positions TNFα not merely as a marker of inflammation but as a key pathophysiological driver of poor outcomes. Additionally, horses with colic often elicit a systemic inflammatory response (SIRS), and a recent study found that horses diagnosed with SIRS at presentation had significantly increased odds of death (36). This evidence collectively highlights the significant role of TNFα-mediated inflammation in the progression and severity of colic in horses. Mechanistically, TNFα is rapidly released from activated macrophages and damaged intestinal tissues in response to ischemia and endotoxemia, where it promotes vascular permeability, leukocyte recruitment, and shock-like responses; speculatively, this early surge may set the stage for multi-organ dysfunction, thereby explaining its strong correlation with poor outcomes in equine colic.

Taken together, these findings suggest that *A. muciniphila* represents a biologically plausible candidate of interest for future investigation in equine colic, based on shared inflammatory and barrier-related pathways observed across species. By promoting anti-inflammatory signaling and attenuating key mediators, such as IL-6 and TNF-*α*, both of which are strongly associated with poor outcomes in colic. *A. muciniphila* could help restore immune balance and reduce the severity of systemic inflammatory responses in affected horses. While current evidence is largely derived from human and rodent studies, the overlap in inflammatory pathways suggests a promising avenue for future equine research, in which modulation of the gut microbiota, including targeted enrichment of *A. muciniphila*, may represent a novel adjunctive strategy to mitigate inflammation and improve survival in horses with colic.

Taken together, these immune pathways form an interconnected mechanistic cascade linking microbiome disruption to clinical outcomes in equine colic. Loss of barrier-protective microbes such as *A. muciniphila* contributes to epithelial permeability, facilitating translocation of luminal endotoxins and activation of innate immune cells. This triggers early release of pro-inflammatory cytokines, particularly TNF-*α* and IL-6, which amplify local tissue injury, vascular permeability, and systemic inflammatory responses. In parallel, disruption of tolerogenic signaling, normally mediated through TGF-*β*–dependent induction of regulatory T cells, reduces immune restraint, allowing unchecked inflammation to persist. The combined effect of elevated TNF-α and IL-6 alongside diminished TGF-β/Treg activity perpetuates a cycle of barrier breakdown, endotoxemia, and dysregulated motility, ultimately manifesting as increased colic severity, systemic inflammatory response syndrome, and poorer clinical outcomes. This integrated framework highlights how cytokine imbalance and barrier dysfunction act synergistically rather than independently in the pathophysiology of equine colic.

Amuc_1100, *A. muciniphila* outer membrane protein, plays a crucial role in improving gut barrier integrity and reducing inflammation by directly interacting with the host immune system (39–42). Its primary mechanism involves binding to Toll-like receptor 2 (TLR2) on the surface of intestinal epithelial cells and immune cells (43). This interaction triggers a signaling cascade that strengthens the tight junctions between epithelial cells, thereby reducing intestinal permeability and preventing the leakage of pro-inflammatory microbial components, such as lipopolysaccharides (LPS), into the bloodstream, a process often called “leaky gut” (44). Furthermore, this TLR2 activation promotes a state of immunological tolerance, modulating the immune response to shift away from a pro-inflammatory state and toward an anti-inflammatory one (45). This is characterized by reduced pro-inflammatory cytokines and increased anti-inflammatory signals. Consequently, by fortifying the gut barrier and calming the local immune environment, Amuc_1100 effectively breaks the vicious cycle of permeability and inflammation that underpins many gastrointestinal and metabolic diseases (41).

In horses, colic, particularly strangulating intestinal obstructions, is a primary cause of increased intestinal permeability, or “leaky gut,” which can have devastating systemic consequences (46). Pathogenesis involves intestinal ischemia–reperfusion injury, where the initial strangulation causes hypoperfusion and ischemic damage to the metabolically active intestinal epithelium (47, 48). Upon restoration of blood flow, reperfusion injury occurs, generating an influx of reactive oxygen species (ROS) that cause lipid peroxidation and direct damage to cellular membranes and tight junctions, critically compromising the epithelial barrier (49, 50). This breach allows for the translocation of luminal contents, including bacterial lipopolysaccharides (LPS) and pathogens, into the systemic circulation (51). The resulting endotoxemia triggers aSIRS and can lead to multiple organ dysfunction syndrome and death, which is why survival rates for ischemic/strangulating lesions are lower than for simple obstructions (52, 53). Furthermore, dietary factors associated with colic risk, such as high starch intake, can cause dysbiosis and hindgut acidosis, which also increases intestinal permeability and may predispose horses to endotoxemia and laminitis (54, 55). Thus, the cycle of permeability, inflammation, and systemic illness is a central pathophysiological mechanism in the most severe forms of equine colic.

Taken together, these insights highlight the potential of *A. muciniphila*, and specifically its outer membrane protein Amuc_1100, as a novel modulator of the gut barrier in equine colic. By directly enhancing epithelial integrity through TLR2 activation and reducing intestinal permeability, *A. muciniphila* may counteract the “leaky gut” cascade that drives endotoxemia, SIRS, and poor outcomes in horses with ischemic or strangulating lesions. Given that barrier dysfunction and inflammation are central to the pathogenesis of severe colic, strategies to enrich *A. muciniphila* within the equine gut microbiota could represent a promising adjunctive approach to mitigate disease severity, improve survival, and reduce long-term complications.

A study by Park et al., found that the genus *Akkermansia* was significantly less abundant, while lactic acid-producing bacteria and streptococci were significantly more abundant in horses with small intestinal colic compared to their healthy counterparts (15). This decrease in *Akkermansia* and the overgrowth of lactic acid-producing bacteria and streptococci contribute to a less diverse and stable gut community, which is associated with the disease state (15). The overgrowth of lactic acid-producing bacteria and streptococci in horses presenting with colic leads to a lower hindgut pH, which likely creates an unfavorable environment for acid-sensitive bacteria such as *Akkermansia*, further disrupting normal fermentation processes and potentially contributing to the development of colic (15).

**Figure 2:**
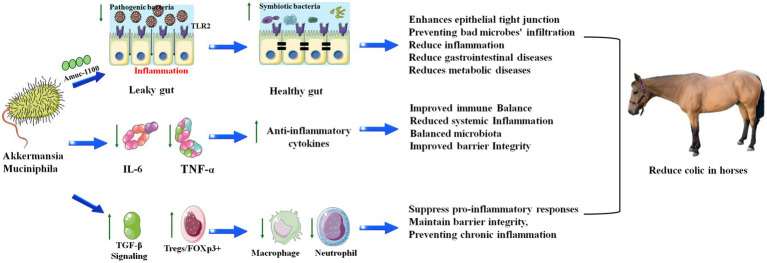
Key functions of *Akkermansia muciniphila* in regulating host metabolic pathways. *A. muciniphila* exerts beneficial effects on the gut through several molecular mechanisms. Its outer membrane protein, Amuc_1100, activates TLR2 signaling, thereby strengthening epithelial tight junctions and reducing barrier permeability. In parallel, *A. muciniphila* decreases pro-inflammatory cytokines, such as IL-6 and TNF-*α*, while promoting the production of anti-inflammatory cytokines. It also activates the TGF-*β* pathway, thereby enhancing the activity of regulatory T cells (Treg/FOXP3^+^), which suppresses the overactivation of macrophages and neutrophils. Together, these actions reduce inflammation, maintain mucosal barrier integrity, and contribute to metabolic homeostasis.

The link between this immunoregulatory mechanism and colic, particularly certain forms of the disease, lies in the disruption of the precise immune-microbial homeostasis within the equine gut (7). Colic is often a consequence of intestinal dysfunction, which can be triggered or exacerbated by low-grade inflammation, dysbiosis (microbial imbalance), and a compromised gut barrier (7). In a healthy state, bacteria like *Verrucomicrobia* (genus *Akkermansia*) promote immune tolerance by inducing regulatory T-cells (Tregs) through a process mediated by tolerogenic dendritic cells and the key anti-inflammatory cytokine transforming growth factor-beta (TGF-*β*) (16, 22). This TGF-β rich environment drives the development of Foxp3 + Tregs, which subsequently suppress pro-inflammatory responses and maintain barrier integrity, thereby preventing a state of chronic inflammation (16). However, a diet high in starch or other stressors can cause a dramatic shift in the microbial population, leading to a decline in these beneficial, immunoregulatory bacteria (56). As their abundance diminishes, this critical signaling pathway is disrupted: the induction of Tregs is reduced, and the suppressing influence on the immune system is lost (56). This allows unchecked activation of pro-inflammatory pathways, leading to localized inflammation of the gut wall. This inflammation can disrupt normal motility, alter secretion, and compromise the mucosal barrier, potentially leading to pain, gas accumulation, and the onset of colic symptoms (57). Furthermore, a weakened barrier allows bacteria and toxins to translocate into the bloodstream, triggering a systemic inflammatory response that can severely worsen the colic episode (58, 59). Therefore, the loss of these specific bacterial groups is not just a symptom of dysbiosis but may also be a fundamental cause of the immune dysregulation that predisposes the intestine to functional disturbances manifesting as colic (Figure 2).

## 4 Prebiotics as a strategy to enhance *Akkermansia*

Evidence from non-equine models indicates that *A. muciniphila* abundance can be enhanced by certain dietary interventions, particularly those involving fermentable fibers and polyphenol-rich plant compounds (60). However, in adult horses, direct evidence supporting prebiotic-driven enrichment of this bacterium is currently lacking. Polyphenols from berries, grapes, and tea have been shown to selectively encourage *A. muciniphila* growth (depending on dosage), possibly through microbial biotransformation products that act as signaling molecules or secondary substrates (61, 62). Grape proanthocyanidin, chlorogenic acid, and resveratrol are notable examples of polyphenols with anti-inflammatory effects in the host and have been associated with increased proliferation of *A. muciniphila* (63, 64). In mice, grape proanthocyanidin has been shown to promote intestinal colonization by *A. muciniphila in vivo*, but not to increase its abundance *in vitro* (36694591). This discrepancy likely reflects the fact that grape polyphenols require host-mediated metabolism, microbial co-metabolism, or mucin induction to generate substrates that support *A. muciniphila* expansion, processes that do not occur in simplified in vitro culture systems. *Bofutsushosan* (BFT), a traditional East Asian herbal remedy, has been shown to decrease inflammatory gene expression in obese mice (65). BFT treatment decreased *Bacteroidetes* and increased Verrucomicrobiota, leading to a bloom of *A. muciniphila*. Inulin-type fructans and FOS may also contribute indirectly by supporting microbial communities that produce mucin-inducing metabolites, thereby improving conditions for *A. muciniphila* proliferation (66).

Notably, equine studies investigating prebiotic supplementation have not been designed to evaluate *A muciniphila* as a primary outcome. Instead, most equine trials assess global microbiome composition, short-chain fatty acid production, immune parameters, or clinical endpoints. While prebiotics can modulate microbial stability and host responses, species-specific effects on *A. muciniphila* abundance in adult horses have not been systematically examined.

In horses, the practical application of these findings will require careful balancing. While fermentable fibers can benefit microbial diversity and SCFAs production, their misuse carries meaningful risks in horses. Excessive doses, rapid introduction, or use during periods of reduced motility can lead to gas overproduction, hindgut acidosis, and distention all of which may precipitate clinic colic, potentially triggering discomfort or colic in sensitive individuals (7). Additionally, baseline microbiota composition varies widely between horses, meaning that some may respond robustly to prebiotic interventions while others show minimal change (7). Identifying which horses are most likely to benefit from supplementation and tailoring it accordingly will be essential for maximizing therapeutic impact while minimizing risk. Because equine fermentation dynamics amplify small errors in dosing, prebiotic strategies must include careful titration, monitoring, and individualized assessment to avoid adverse events.

At present, no equine trials have systematically quantified the dose at which prebiotic-associated benefits outweigh fermentative risk, reinforcing the need for controlled dose-escalation studies incorporating objective endpoints such as hindgut pH, SCFA profiles, gas production, and clinical colic outcomes.

## 5 Clinical evidence and translational insights

To ensure transparency regarding clinical readiness, the evidence supporting *A. muciniphila*–based interventions in equine colic can be stratified into three categories. First, direct equine clinical intervention studies evaluating *A. muciniphila* supplementation or targeted prebiotic modulation are currently absent. Second, equine observational studies have reported associations between intestinal disease states and altered *A. muciniphila* abundance; however, these studies are cross-sectional and do not establish causality. Third, mechanistic evidence derived from non-equine models, including rodent and human studies, demonstrates that *A. muciniphila* influences mucosal barrier integrity, immune modulation, and inflammatory signaling pathways relevant to colic pathophysiology. Accordingly, translational relevance in horses is presently supported by biological plausibility and associative equine data rather than direct clinical efficacy.

Direct intervention trials examining *A. muciniphila* in horses do not yet exist, but indirect evidence supports its potential role in colic prevention. Accordingly, all translational interpretations in this section should be viewed as hypothesis-supporting rather than confirmatory, and no causal relationship between prebiotic supplementation, *A. muciniphila* modulation, or colic prevention in horses can currently be inferred.

Studies have shown that prebiotics like MOS and FOS can improve microbial stability during dietary changes, reduce fecal shedding of pathogenic bacteria, and enhance SCFAs production, all outcomes associated with a healthier hindgut environment (67).

In humans and rodent models, both pasteurized *A. muciniphila* supplementation and indirect dietary strategies that increase its abundance have improved gut barrier integrity and attenuated systemic inflammation (68). These host-protective effects mirror the physiological vulnerabilities seen in horses prone to colic, especially those exposed to abrupt dietary changes, high-starch feeding, or environmental stress. Additionally, certain probiotics (e.g., *Lactobacillus fermentum*, *Bacillus subtilis*) have been shown to reduce colitis-associated inflammation while secondarily increasing *A. muciniphila* abundance, highlighting a potential multifaceted route to microbial modulation (62, 69). These effects align closely with the physiological needs of horses at risk for colic, particularly those exposed to high-grain diets, restricted forage, or frequent environmental stressors.

An additional translational opportunity lies in diagnostics. If *A. muciniphila* abundance inversely correlates with inflammatory markers or colic incidence in horses, it could serve as a valuable biomarker for early risk detection. It was shown that feeding mice soy protein isolate-sorbed Concord grape polyphenols increased the growth of *A. muciniphila* while inhibiting the growth of *Firmicutes* and *Bacteroidetes* (61). The development of equine-specific quantitative assays, such as targeted qPCR or digital droplet PCR for *A. muciniphila* quantification, metagenomic abundance profiling, and ELISAs measuring mucin-related barrier markers (e.g., MUC2), LPS-binding protein, or inflammatory cytokines- would allow veterinarians to monitor gut health with far greater precision and intervene before clinical signs of colic appear.

From a translational perspective, feasibility and regulatory considerations will strongly influence the adoption of microbiome-based therapies in horses. Live biotherapeutics, including targeted *A. muciniphila* preparations, would likely fall under veterinary biologics oversight and require safety, purity, and efficacy testing. Additionally, production costs, storage requirements, and the need for strain-specific stability data may limit availability. Economic analyses and regulatory pathways should be defined early to ensure realistic clinical application.

A “leaky” gastrointestinal barrier primarily contributes to medical forms of colic, including inflammation-associated colic, dysmotility, hindgut acidosis, and immune-mediated gastrointestinal disorders (7, 46). Barrier dysfunction can increase visceral sensitivity, impair motility, and promote gas accumulation, thereby predisposing horses to spasmodic colic or recurrent low-grade colic (7). In these contexts, increased permeability allows translocation of bacterial products such as LPS and amplifying inflammation (23, 70).

This mechanism, however, is fundamentally different from the pathophysiology of surgical colic, where the initiating event is a mechanical obstruction (e.g., volvulus, strangulating lipoma, entrapment, mesenteric rent) (71). Surgical lesions cause rapid, localized ischemia independent of microbiota status or barrier integrity (72, 73). Although a compromised barrier or dysbiosis may worsen the systemic inflammatory response after ischemic injury, they do not precipitate the obstructive event itself (6). It is important to emphasize that equine colic is not a single disease entity but a collection of syndromes with diverse etiologies. While dysbiosis and intestinal barrier dysfunction may contribute to certain medical or inflammatory forms of colic, they may also arise secondary to colic-associated ischemia, stress, dietary disruption, or therapeutic interventions. Consequently, the temporal relationship between dysbiosis and colic remains unclear, and current evidence does not support a unidirectional causal model.

During surgical colic, reperfusion following correction of the obstruction causes mucosal breakdown, which permits endotoxin and bacteria to enter the circulation, driving endotoxemia and SIRS, the hallmark of severe surgical colic (36, 74, 75).

Therefore, the potential role of *A. muciniphila*, prebiotics, and other microbiome-directed therapies should be contextualized within the medical colic setting. These interventions may enhance mucosal resilience, stabilize fermentation, and reduce inflammation, thereby lowering the risk of functional gastrointestinal disturbances. They cannot prevent mechanically driven surgical colic, although they may influence postoperative recovery or modulate systemic inflammation secondary to strangulating lesions.

## 6 Future directions

A key next step is to establish the baseline abundance and distribution of *A. muciniphila* in healthy versus colic-prone horses, while accounting for influential factors such as diet, age, season, and training level. This foundational data will guide hypothesis-driven intervention trials to modulate its levels through prebiotic or synbiotic supplementation.

As previously described in microbiome-modulation literature, Synbiotic, which combines a targeted probiotic with a complementary prebiotic, may offer a particularly effective way to promote *A. muciniphila* and its functional benefits (76, 77). This approach has shown promise in other species, where the dual action of introducing beneficial microbes while supplying their preferred substrates creates a more durable shift in the microbiome. A few equine-specific synbiotic studies exist. For example, Lagounova et al. demonstrated that a synbiotic (PROBIOPlus™) administered to actively racing Standardbred horses during antibiotic therapy altered the hindgut microbial community composition and fiber-degrading taxa (78). MacNicol et al. conducted an *in vitro* equine cecal and fecal-slurry fermentation model and found that a probiotic/prebiotic supplement increased production of acetate, propionate and butyrate, although microbial composition changes were limited (26). For a broader foundational context, Markowiak and Śliżewska review synbiotics in livestock nutrition and outline criteria and evidence for their use (79). For equine applications, synbiotic formulations could be tailored to the unique hindgut fermentation environment, potentially using plant-based polyphenols alongside select oligosaccharides.

Another research gap lies in dosing strategies. In horses, the fermentative capacity of the hindgut means that subtle changes in substrate availability can produce disproportionate shifts in gas production and pH. Establishing optimal dosages and delivery methods will be crucial for ensuring efficacy without adverse effects. Future research should also assess alternative dosing and delivery methods for horses experiencing colic episodes, if synbiotics prove effective at that time.

In addition to probiotics and prebiotics, fecal microbiota transplantation (FMT) has emerged as another microbiota-modifying approach in horses (80). Several clinical reports demonstrate that FMT can improve fecal consistency, restore microbial diversity after antimicrobial-associated colitis, and shorten recovery time compared with probiotic supplementation alone (81). While protocols vary, FMT appears to provide broader microbial restoration than single-strain or limited-strain probiotics, and future work may clarify whether FMT improves outcomes in colic or barrier-related disorders.

At present, the translational readiness of *A. muciniphila*–based strategies in horses should be considered exploratory. While mechanistic studies in non-equine systems provide compelling insights into immune regulation and barrier function, and equine observational studies suggest disease-associated microbial shifts, no controlled equine trials have evaluated safety, dosing, or clinical efficacy. As such, these interventions should be viewed as candidates for future hypothesis-driven investigation rather than clinically deployable therapies.

Building on these future directions, several testable hypotheses can be articulated to guide feasible and biologically informed equine studies. A primary hypothesis is that prebiotic or synbiotic supplementation increases the relative abundance of *A. muciniphila* in horses predisposed to medical colic and is accompanied by measurable reductions in systemic and intestinal inflammatory markers, including IL-6 and TNF-*α*, alongside enhancement of regulatory pathways mediated by TGF-*β* and regulatory T cells.

A second hypothesis is that increases in *A. muciniphila* abundance are associated with improved intestinal barrier integrity, reflected by reduced epithelial permeability, lower endotoxin translocation, and attenuation of systemic inflammatory responses. A third, clinically oriented hypothesis is that horses demonstrating favorable microbiome and immune modulation exhibit reduced severity, duration, or recurrence of medical colic compared with non-responders or untreated controls.

From a feasibility standpoint, early-phase studies should prioritize short-term, hypothesis-driven designs in horses with medical colic or colic predisposition, incorporating serial fecal microbiome profiling, targeted cytokine measurements (e.g., IL-6, TNF-*α*), and standardized clinical outcome metrics. More complex dose-escalation trials, barrier-function assays, and long-term recurrence studies can subsequently be pursued once safety and biological signals are established. Framing future work in this stepwise manner strengthens translational rigor while remaining aligned with the ethical and logistical constraints of equine research.

## 7 Conclusion

Prebiotics offer a promising, biologically grounded means of supporting equine gastrointestinal health and reducing colic risk by fostering beneficial microbial populations, enhancing SCFAs production, and strengthening the gut barrier. Within this context, *A. muciniphila* stands out as a bacterium of high potential interest. Its mucin-degrading niche, ability to stimulate mucus renewal, and demonstrated anti-inflammatory properties position it as both a possible biomarker and a therapeutic target in horses.

While current evidence for *A. muciniphila* in equine health is extrapolated from other species, the consistency of its beneficial effects across models suggests that it deserves focused investigation in horses. *A. muciniphila* abundance could serve as a noninvasive biomarker through fecal or rectal swab quantification, reflecting gut barrier integrity and colic risk, while serum-based adjuncts (e.g., SCFAs, cytokine correlations, or other downstream immune markers) may enhance diagnostic and prognostic precision in clinical settings. Because Amuc_1100–TLR2 signaling is detectable only through cellular or molecular assays, it is unlikely to serve as a practical clinical biomarker; instead, indirect serum indicators of barrier dysfunction and inflammation would be more clinically applicable. The future of colic prevention may well lie in integrating microbiome-based diagnostics with personalized nutritional interventions, approaches that could transform preventive care from a reactive to a proactive discipline.

This review is intentionally framed as a hypothesis-generating synthesis rather than a definitive assessment of therapeutic efficacy. Its primary contribution lies in defining biologically plausible mechanisms, contextualizing limited equine observations, and outlining a rationale for future controlled experimental and clinical studies.

Current evidence does not support a colic-specific role for *A. muciniphila* independent of broader microbiome alterations, and its observed changes should be interpreted within the context of global microbial community shifts.

Additionally, no studies to date demonstrate that prebiotic supplementation increases *A. muciniphila* abundance in horses, highlighting the need for age-specific, hypothesis-driven equine intervention studies.

Future translational progress will depend on rigorously designed equine studies that define safe and effective dosing ranges for prebiotic interventions using quantitative fermentation and clinical outcome measures.
